# Reinforcing the Functionality of Mononuclear Phagocyte System to Control Tuberculosis

**DOI:** 10.3389/fimmu.2018.00193

**Published:** 2018-02-09

**Authors:** Susanta Pahari, Gurpreet Kaur, Shikha Negi, Mohammad Aqdas, Deepjyoti K. Das, Hilal Bashir, Sanpreet Singh, Mukta Nagare, Junaid Khan, Javed N. Agrewala

**Affiliations:** ^1^Immunology Laboratory, CSIR-Institute of Microbial Technology, Chandigarh, India

**Keywords:** mononuclear phagocyte system, tuberculosis, monocyte, macrophage, dendritic cell, pattern recognition receptors, infection, immunotherapy

## Abstract

The mononuclear phagocyte system (MPS) constitutes dendritic cells, monocytes, and macrophages. This system contributes to various functions that are essential for maintaining homeostasis, activation of innate immunity, and bridging it with the adaptive immunity. Consequently, MPS is highly important in bolstering immunity against the pathogens. However, MPS is the frontline cells in destroying *Mycobacterium tuberculosis* (*Mtb*), yet the bacterium prefers to reside in the hostile environment of macrophages. Therefore, it may be very interesting to study the struggle between *Mtb* and MPS to understand the outcome of the disease. In an event when MPS predominates *Mtb*, the host remains protected. By contrast, the situation becomes devastating when the pathogen tames and tunes the host MPS, which ultimately culminates into tuberculosis (TB). Hence, it becomes extremely crucial to reinvigorate MPS functionality to overwhelm *Mtb* and eliminate it. In this article, we discuss the strategies to bolster the function of MPS by exploiting the molecules associated with the innate immunity and highlight the mechanisms involved to overcome the *Mtb*-induced suppression of host immunity. In future, such approaches may provide an insight to develop immunotherapeutics to treat TB.

## Introduction

Despite of the fact that efficient anti-tuberculosis (TB) drugs are available, TB remains to ruin public health globally. Reports suggest that one-third of the populace is infected with *Mycobacterium tuberculosis* (*Mtb*), almost 10.4 million active cases and around 1.8 million deaths in 2016 (1). The occurrence of threat is further complicated due to acquired immunodeficiency syndrome pandemic, the appearance of multidrug-resistant (MDR), extensively drug-resistant, as well as totally drug-resistant *Mtb* strains (2). Vaccines are the most effective strategy to control and eliminate any disease (3, 4). Ironically, bacillus Calmette–Guérin (BCG) is the most controversial vaccine because of its variable efficacy worldwide (5). Moreover, it protects only children but not adults (6). Therefore, an urgent necessity and the challenge for the scientific society are to improve the current drug regimen or develop alternative stratagems against TB.

Our immune system is quite complex and complicated, comprising of innate as well as adaptive branch of immunity. Innate immunity is the primary and foremost line of defense against intruding pathogens (7). Innate immunity was initially believed to be non-specific and considered to be of lesser importance for the immune function. On the other hand, adaptive immunity is allied with the exclusion of intracellular pathogens in the subsequent stages of infection. It was considered as sentinel of the immune system owing to its specificity as well as immunological memory generation. Since the last few decades, innate immunity has gained enormous consideration due to the discovery of “germ line-encoded” pattern recognition receptors (PRRs), which makes the innate immunity capable of discriminating between self and an array of pathogens (8). PRRs are predominantly expressed by various antigen-presenting cells (APCs) such as monocytes, macrophages, and dendritic cells (DCs). These cells constitute the mononuclear phagocyte system (MPS). Mononuclear phagocyte cells (MPCs) are progenitors derived from bone marrow hematopoietic cell lineage (9). “Committed myeloid progenitor cells” can differentiate into blood monocytes, which then migrate to the bloodstream and subsequently enter in different tissues to develop into the resident tissue macrophages and DCs (10, 11). In the conventional sight of the MPS, cell division happens primarily in monoblasts and promonocytes. The expansion of mature macrophages provides the maintenance and number of resident tissue macrophages (10). MPCs mainly contribute in the recognition and eradication of pathogens and their related products. Furthermore, they contribute substantially in promoting innate immunity and subsequently stimulating, shaping, and expanding the adaptive immunity (12). Initiation of adaptive immunity not only depends on the direct detection of antigen by the receptors of MPCs but also relies on crucial signals delivered through costimulatory molecules, cytokines, and PRRs (13). Importantly, DCs contribute considerably in bridging innate and adaptive immunity (8, 14). DCs express a plentiful amount of costimulatory molecules and PRRs, which regulate several immune functions and signaling cascades that are crucial for the instigation of adaptive immune response (15). In addition, they successively alert other immune cells to accumulate at the infection site. Furthermore, they combat and resist *Mtb* in establishing infection and restrain them from becoming an active disease.

Based on the aforementioned investigations, MPS are considered as an important first line of defense against pathogen. Exploiting MPCs or their components, namely, PRRs, costimulatory molecules, cytokines, and chemokines as therapeutic agents may be an exciting line of study to control TB. Previously, our group has highlighted the importance of signaling through innate molecules in context with nasal and mucosal immunity to restrict *Mtb* entry and consequently prohibiting its infection. We discussed the role of several immunomodulators *in vitro, in vivo*, or in clinical studies to enhance the efficacy of anti-TB drugs in treating TB patients (16). Current review highlights the interaction between *Mtb* and MPS influencing the outcome of disease. Hence, as evidenced by published literature, we hypothesize a crucial strategy to reinvigorate MPS functionality to overwhelm *Mtb* and eliminate it. Furthermore, we discuss the strategies to bolster the function of MPS by exploiting the molecules associated with the innate immunity and highlight the mechanisms involved therein. It may be hypothesized that involving MPS in conjunction with drugs, as an adjunct therapy may lessen the dose as well as duration of ongoing drug regimen; and therefore, may reduce the chances of developing drug resistance by the pathogen.

## Various Mononuclear Phagocytic Cells and Their Function in Innate and Adaptive Immunity

Mononuclear phagocyte cells located in various tissues differ in terms of their nomenclature and morphological appearance (17). For example, macrophages are called as histiocytes in subcutaneous tissues, Kupffer cells resides in liver, microglia present in nervous tissue, alveolar macrophages in lungs, osteoclasts in bones, etc. Besides phagocytosing pathogens and eliminating them from the blood, lymph, and tissues, MPS also clears the senescent cells and mounts immunity against the pathogens (18). MPS recognizes, captures, and internalizes the pathogenic determinants identified as pathogen-associated molecular patterns (PAMPs) through PRRs localized on their surface. This leads to the secretion of biologically active molecules such as free radicals, cytokines, and chemokines. The chemokines attract chiefly neutrophils from the bloodstream and initiate a pro-inflammatory response leading to the engulfment and destruction of *Mtb*.

Lung alveolar macrophages and myeloid DCs are some of the foremost cell types that get infected after aerosol challenge with *Mtb*. Subsequently, interstitial macrophages, monocytes, and neutrophils are recruited to infection site (19). MPCs capture *Mtb* and migrate to the local draining lymph nodes, then process and present antigens efficiently in context with MHCs to activate T cells (20). The intensity of MPCs and *Mtb* counterattack widely depends upon the host genetics as well as bacterial virulence factors. Accordingly, *Mtb* replicates within the host MPCs (21, 22) and manipulates function by impairing their ability to control infection (23, 24). *Mtb* can obstruct the antigen processing and presentation by MPCs to T cells (25–27). Macrophages and DCs that are not optimally activated cannot kill the intracellular *Mtb* and serve as a reservoir for the dissemination of the pathogen. In addition, due to their striking migratory potential, they play a key role in transmitting *Mtb* from the site of infection to other tissues (28).

## Evasion Strategies Adopted by *Mtb* to Counteract Host Immunity

One of the major mechanisms through which *Mtb* obstructs MPS function is by inhibiting the fusion of phagosome with lysosome. Various mycobacterial lipids and glycolipids, proteins, and enzymes, namely, lipoarabinomannan (LAM) and trehalose-6,6’-dimycolate (TDM), protein tyrosine phosphatase A (PtpA), secretory acid phosphatase M, zinc-dependent metalloprotease 1, lipoamide dehydrogenase C, serine/threonine protein kinase G, and PEPGRS62 protein have been proved to play an important role in the capacity of *Mtb* to escape phagolysosome fusion (29–32).

Two signals are important for the optimal activation of T cells. Initial one is the engagement of TCR with MHC–peptide complex and subsequent upregulation of costimulatory molecules. Instead of getting activated, T cells get anergized (“a state of unresponsiveness”) in the absence of costimulatory molecules (33). Interestingly, *Mtb* has the ability to successfully down modulate the expression of costimulatory molecules. Furthermore, after infecting MPCs, *Mtb* upregulates the expression of immunosuppressive markers programmed cell death-1, Lymphocyte activation gene-3, and T-cell immunoglobulin mucin-domain containing-3, thus retaliating against the potential threat caused by T cells (34, 35).

Another mechanism is the deprivation of MPS nutrients by *Mtb*. The most common battle between host cells and the pathogen is for iron utilization. *Mtb* efficiently utilizes its siderophores for iron uptake and thus deprives host of its availability (36). Furthermore, carbon from various sugars and fatty acids are extracted by *Mtb* in host cells *via* its major enzymes, such as the polyphosphate glucokinase, isocitrate lyases (ICL1 and ICL2), and the phosphoenolpyruvate carboxykinase (37–39). *Mtb* favors the differentiation of macrophages toward M2 subtype (40). By contrast, it impairs the formation of M1 macrophages. M2 macrophages are responsible for suppression of inflammatory function. M1 subtype arises from type-1 inflammatory conditions and secretes pro-inflammatory cytokines and is endowed with microbicidal activity (41). Virulence factors of *Mtb* are known to preferably skew the generation of M2 macrophages (42). Therefore, *Mtb* is successful in creating an environment for its intracellular survival inside macrophages (43, 44). Furthermore, *Mtb* mainly skews the differentiation of CD4 T cells toward Th2 cells phenotype (45). Similarly, the generation of regulatory T cells that secrete TGF-β is promoted. Both Th2 cells and Tregs help in the TB progression. By contrast, the formation of Th1 cells and Th17 cells is suppressed by hijacked MPS, since they have potential to successfully control the *Mtb* infection (46).

## MPS Helps in the Restoration of Host Immunity Impaired by *Mtb*

Mononuclear phagocyte system contributes significantly to the health and disease (47). One of the most imperative mechanism and early response of innate immunity is the generation of reactive oxygen species (ROS) by MPS, which not only destroys the pathogen but also plays a physiological role in maintaining and controlling the cellular functions. Clearance of colonized microorganisms and initiation of signaling pathways related to inflammation, cell proliferation, and induction of immunity is highly dependent on ROS (48). Two sources of ROS generation in the host upon microbial infection is membrane-bound NADPH oxidase complex as well as mitochondrial electron transport chain (49). Important PRRs associated with an intracellular pathogen is NOD-like receptors (NLRs), which makes cell attentive on pathogen interaction/invasion. Among many NLRs, NLRX1 moves to mitochondria and initiates the ROS production (50).

## Role of MPS to Overcome the Modulation of Cellular Metabolism and Nutrient Acquisition by *Mtb*

*Mycobacterium tuberculosis* utilizes cholesterol for its survival and establishes infection in the host cells (43). This cholesterol is further converted into sterol, which is crucial for *Mtb* persistence in the host cells (51). Adenosine-5′-triphosphate (ATP) plays a decisive role in the host by acting directly on cell metabolism and signaling cascade. The ATP that comes out of the cell into the extracellular environment is known as extracellular ATP (eATP). It has been seen that eATP can activate the immune system by acting as a “danger signal” (52). Moreover, it is well known that eATP has a potential role in stimulating the release of pro-inflammatory cytokines. eATP induces IL-6 secretion from macrophages (53) and IL-1β production from LPS primed monocytes (54). Furthermore, it is noted that eATP signaling is not only implicated in the generation of ROS and pro-inflammatory cytokines but also plays a significant role in antigen presentation. Previous report demonstrated that eATP along with its putative receptor P2X7 on inflammasome activation induces the shedding of exosomes containing the MHC class II from macrophages (55). It is well-known fact that ROS signaling is involved in the inflammasome formation (56). Thus, it facilitates innate as well as adaptive immune response. *Mtb*-infected phagocytes release exosomes containing the MHC class II and *Mtb* Ag85B, which activates the T cells (57). The eATP–P2X7 receptor signaling plays an important role in clearing *Mtb* infection through multiple ways such as phospholipase-D (58), apoptosis (57), phagosome–lysosome fusion (59), and autophagy (60). Thus, MPS is recognized to play an appreciable role in neutralizing and eradication of *Mtb* from the host.

## Involvement of Phagocytic Cells to Boost Immunity Against *Mtb*

Both DCs and macrophages play crucial roles in protection against mycobacterium. The presence of *Mtb* at infection site is sensed by macrophages through chemokine-mediated migration, as these macrophages express surface receptor for these chemokines known as G-protein-coupled receptors (61). *Mtb* is efficiently phagocytosed by these professional phagocytic cells. Studies on human macrophages have shown that phagocytosis is significantly improved in the presence of anti-*Mtb* antibodies and complement factors (62). Once *Mtb* is phagocytosed by the macrophage or DCs, it encounters a number of defense mechanisms operated through the innate immunity of the host. These include the formation of free radicals, namely, ROS, reactive nitrogen intermediates (RNI), cytokines, and chemokines. Moreover, MPS helps in the differentiation of T cells; DCs secrete IL-12, which results in the generation of Th1 cells. Moreover, Th1 cells mainly secrete IFN-γ that activates macrophages to release TNF-α (63). Similarly, IL-6 and TGF-β secreted by MPS helps to differentiate naïve T cells into Th17 phenotype (64). Th1 cells and Th17 cells can reciprocally regulate the function of Th2 cells and Tregs, respectively. Both Th2 cells and Tregs promote the progression of TB.

Mononuclear phagocyte system employs factors that are involved in basic metabolism of the body to fight against intracellular pathogens. One such important molecule is vitamin D3, which enhances the phagocytosis of MPS by upregulating the expression of CD14 and CD206 receptors (65). Toll-like receptor (TLR)-2 signaling in macrophage upregulates the expression of vitamin-D–1-hydroxylase and surface vitamin-D receptor, which stimulates the generation of antimicrobial peptide cathelicidin and contributes to resistance to *Mtb* (66).

## PRRs-Mediated Bolstering of MPS Activity Against *Mtb*

Mononuclear phagocyte cells are the key sensory cells that reinforce the innate immunity. They express the plethora of innate receptors such as TLRs, NLRs, and C-type lectin receptors (CLRs), which are collectively called as PRRs that are present either on the cell surface or endocytic vesicles. The PRRs including TLRs (TLR-2, -3, -4, and -9) and non-TLRs [CLRs, NLRs such as nucleotide-binding oligomerization domain (NOD)-2, mannose receptors (MRs), Dectin-1, and DC-SIGN] recognize conserved PAMPs that are present on *Mtb*. PRRs have the capacity to recognize a broad range of structural components of pathogens grouped as PAMPs and DAMPs, which includes lipopeptides, lipoproteins, lipoteichoic acid, peptidoglycans, ssRNA, dsRNA, siRNA, mRNA, DNA, LPS, heat shock proteins, and flagellin. The role of several PPRs in protecting against *Mtb* has been widely studied (16, 67–70). The interaction of PRRs with PAMPs triggers a series of signaling pathways inside the MPCs (Table 1). PRRs activated MPCs acquire augmented expression of MHC I, MHC II, and costimulatory molecules on their surface (63), which leads to the better presentation of *Mtb* antigens to naïve T cells followed by generation of efficient T cell response against this pathogen. *Mtb* loaded macrophages, after activation, can mount bactericidal activities such as nitric oxide (NO) production, maturation of phagosomes toward phagolysosomes and autophagolysosomes (71). Recruitment and activation of many signaling molecules in cascade lead to nuclear translocation of NF-κB, which eventually causes the activation of MPCs. In a different setup, the activated macrophages have the capability to carry out macro-autophagy to take care of intracellular *Mtb* (72, 73). In a similar phenomenon known as “programmed necroptosis,” MPCs controls the intracellular replication of *Mtb*. This process speeds up the recruitment of neutrophils and thereby enhances the killing of mycobacterium (74). MPS activation is evident by the release of pro-inflammatory cytokines such as IL-1β, IL-6, IL-12, and TNF-α; which help in phagocytosis of the bacterium followed by activation of *Mtb* reactive T cells. These T cells play a cardinal role in controlling the *Mtb* growth.

**Table 1:** Activation of PRRs through PAMPs.

	PRRs (structure)	Adapters (structure)	PAMPs/activators	Species	Cell types	Location
**TLR**	TLR-1–TLR-2 (LRR-TIR)	MyD88 (TIR-DD) and TIRAP (TIR)	Triacyl lipopeptides	Bacteria	Granulocytes, macrophages, mDCs, monocytes, and B cells	Cell surface

	TLR-2–TLR-6 (LRR-TIR)	MyD88 and TIRAP	Diacyl lipopeptides	Mycoplasma	Granulocytes, macrophages, mDCs, monocytes, and B cells	Cell surface
			LTA	Bacteria		
			Zymosan	Fungus		

	TLR-2 (LRR-TIR)	MyD88 and TIRAP	PGN	Bacteria	Granulocytes, macrophages, mDCs, monocytes, mast cells, and neutrophils	Cell surface
			Lipoarabinomannan	Mycobacteria		
			Porins	Bacteria (Neisseria)		
			tGPI-mucin	Parasites (Trypanosoma)		
			HA protein	Viruses (Measles virus)		

	TLR-3 (LRR-TIR)	TRIF (TIR)	dsRNA	Viruses	DCs, macrophages, NK cells, and B cells	Endosome

	TLR-4 (LRR-TIR)	MyD88, TIRAP, TRIF. TRAM (TIR)	LPS	Bacteria	DCs. macrophages, B cells, monocytes, neutrophils, granulocytes, and regulatory T cells	Cell surface
			Envelope proteins	Viruses (RSV, MMTV)		

	TLR-5 (LRR-TIR)	MyD88	Flagellin	Bacteria	Monocytes, DCs, mast cells, epithelial cells, mast cells, and regulatory T cells	Cell surface

	TLR-7 (LRR-TIR)	MyD88	ssRNA	RNA viruses	B cells, DCs, macrophages, monocytes, and regulatory T cells	Endosome

	hTLR-8 (LRR-TIR)	MyD88	ssRNA	RNA viruses	Monocytes, DCs, mast cells, epithelial cells, mast cells, and regulatory T cells	Endosome

	TLR-9 (LRR-TIR)	MyD88	CpG DNA	Bacteria	DCs. macrophages, B cells, monocytes, and neutrophils	Endosome
			DNA	DNA viruses		
			Malaria hemozoin	Parasites		

	TLR-10	Unknown	Unknown	Unknown	B cells, monocytes, neutrophils, and pDCs	Cell surface

	mTLR-11 (LRR-TIR)	MyD88	Not determined	Bacteria (uropathogenic bacteria)	Monocytes, macrophages, and epithelial cells	Endosome
			Profilin-like molecule	Parasites (*Toxoplasma gondii*)		

	TLR-12	MyD88	Profilin-like molecule	Parasites (*Toxoplasma gondii*)	DCs, macrophages, and neurons	Unknown

	TLR-13	MyD88, TAK-1	Bacterial 23S ribosomal RNA (rRNA)	Virus, bacteria	Monocytes, macrophages, and DCs	Endosome

**RLR**	RIG-I (CARDx2-helicase)	IPS-1 (CARD)	RNA (5′-PPPssRNA, short dsRNA)	Viruses	cDCs, macrophages, and fibroblasts	Endosome

	MDA5 (CARDx2-helicase)	IPS-1	RNA (poly IC, long dsRNA)	Viruses	cDCs, macrophages, and fibroblasts	Endosome

	LGP2 (helicase)		RNA	Viruses	cDCs, macrophages, and fibroblasts	Endosome

**NLR**	NOD-1/NLRC1 (CARD-NBD-LRR)	RICK (CARD), CARD9 (CARD)	iE-DAP	Bacteria	DCs, macrophages, and epithelial cells	Endosome

	NOD-2/NLRC2 (CARDx2-NBD-LRR)	RICK, CARD9	MDP	Bacteria	DCs, macrophages, and epithelial cells	Endosome

	NALP3/NLRP3 (PYD-NBD-LRR)	ASC (PYD-CARD) CARDINAL (PYD-FIND)	MDP	Bacteria	DCs, macrophages, epithelial cells, and T cells	Cytoplasm
			RNA	Bacteria and viruses		
			ATP	Bacteria		
				Host		
			Toxins	Bacteria		
			Uric acid, CPPD, amyloid-β	Host		

	NALP1/NLRP1 (CARD-FIND-NBD-LRR-PYD)	ASC	Anthrax lethal toxin	Bacteria	Bone marrow blast cells, epithelial cells, Langerhans cells, and neurons	Cytoplasm

	IPAF/NLRC4 (CARD-NBD-LRR)		Flagellin	Bacteria	Hematopoietic cells, macrophages, and epithelial cells	Cytoplasm

	NAIP5 (BIRx3-NBD-LRR)		Flagellin	Bacteria	Hematopoietic cells, macrophages, and epithelial cells	Cytoplasm

**CLR**	Dectin-1 (lectin-ITAM)		β-Glucan	Fungi, bacteria	DCs, macrophages, monocytes, neutrophils, B cells, and NK cells	Cell surface

	Mincle (Clec4e)	ITAM-bearing adaptor FcRy	TDB and TDM	Mycobacteria and fungi	DCs, macrophages, B cells, and neutrophils	Cell surface

*Distinct signaling cascades are triggered through PRRs against pathogen-associated moieties*.

*The PAMPs expressed on array of pathogens are recognized by the PRRs present on the cells of immune system. The PRRs are located either intracellularly or on the surface of the cells*.

*TLR, toll-like receptor; CLR, C-type lectin receptor; NLR, NOD-like receptor; NOD, nucleotide-binding oligomerization domain; RLR, RIG-like receptor; RIG-1, retinoic acid-inducible gene 1; LRR, leucine-rich repeat receptor; TIR, toll/interleukin-1 (IL-1) receptor; MDA5, melanoma differentiation-associated protein 5; LGP2, laboratory of genetics and physiology 2; NLRC, nuclear oligomerization domain proteins subfamily C; NLRP, NLR family pyrin domain; NBD, nucleotide-binding domains; PYD, pyrin domain; FIND, function to find domain; IPAF, IL-1β-converting enzyme protease-activating factor; NAIP5, neuronal apoptosis inhibitory protein 5; BIR, baculovirus inhibitor of apoptosis protein repeat; ITAM, immunoreceptor tyrosine-based activation motif; Mincle, macrophage-inducible C-type lectin receptor; Clec4e, C-type lectin domain family 4 member e; TIRAP, TIR-domain-containing adaptor protein; MyD88, myeloid differentiation primary response 88; TRIF, TIR-domain-containing adapter-inducing interferon-β; TRAM, TRIF-related adapter molecule; TAK-1, TGF-β-activated kinase 1; IPS-1, interferon promoter stimulator-1; RICK, RIP-like interacting CLARP kinase; CARD, caspase recruitment domain; ASC, apoptosis-associated speck-like protein containing a CARD; TUCAN, tumor-upregulated CARD-containing antagonist of caspase-nine; CARDINAL, CARD8, DACAR, NDPP1, and TUCAN; TDB, trehalose-6,6-dibehenate; TDM, trehalose-6,6′-dimycolate; CPPD, calcium pyrophosphate dihydrate crystals; LTA, lipoteichoic acid; PGN, peptidoglycan; tGPI-mucin, trypomastigote glycosylphosphatidylinositol mucins; HA protein, hemagglutinin protein; LPS, lipopolysaccharides; iE-DAP, d-glutamyl-meso-diaminopimelic acid; MDP, muramyl dipeptide; RSV, respiratory syncytial virus; MMTV, mouse mammary tumor virus; Mdc, myeloid dendritic cells; cDC, conventional dendritic cells; PAMP, pathogen-associated molecular pattern; PRR, pattern recognition receptor; ATP, adenosine-5′-triphosphate; DC, dendritic cell*.

Importance of PRRs signaling in the activation of an immune response against *Mtb* can be accounted by the evidence that MyD88^−/−^ mice were more prone to *Mtb* infection. TLR-2-knockout mice showed low IL-12 and TNF-α yield on *Mtb* infection and more granulomas formation in the lungs (75). The 19 kDa lipoprotein of *Mtb* activates MPCs through TLR-2 triggering and induces IL-12 and NO production, and subsequently killing of *Mtb* (76). In humans, the interaction of 19 kDa lipoprotein with TLR-2 induces apoptosis of *Mtb*-infected macrophages (77). TLR-4 senses HSP60/65 and 38-kDa *Mtb* antigen inducing protective TNF-α production (78). In addition, another *Mtb* small heat shock protein X also recognized as α-crystallin-1 can specifically modulate the function of DCs at different maturation stages (79, 80). TLR-4 activation is known to induce macro-autophagy by recruitment of Beclin-1. Recently, we showed that cumulative signaling of DCs through TLR-4 and NOD-2 successfully inhibits the intracellular survival of *Mtb* through autophagy (70). Mycobacterial DNA interacts with TLR-9 and elicits macrophages to produce pro-inflammatory cytokines. Furthermore, TLR-9-knockout mice showed less release of IFN-γ and IL-12. *Mtb*-infected macrophages and DCs deficient in TLR-9 are less responsive to IL-12 (81). NLRs and CLRs can also influence the function of MPCs. NOD-2-deficient mice exhibit impairment in cytokine and NO release upon *Mtb* infection (82). Activation of *Mtb*-infected human macrophages through NOD-2 induces autophagy and restricts *Mtb* growth (83). Likewise, signaling of *Mtb*-infected DCs through Dectin-1 and macrophage-inducible C-type lectin (Mincle) influences the intracellular survival of the pathogen (84, 85). By contrast, DC-specific intercellular adhesion molecule-3-grabbing non-integrin (DC-SIGN) interaction with LAM of *Mtb* initiates the anti-inflammatory response by inducing the secretion of IL-10 (86). Overall, it signifies that the engagement of various PRRs on MPCs can differentially regulate their function toward *Mtb*.

## Contribution of Inflammatory Response in Controlling *Mtb* Infection

Inflammatory response generated by cytokines helps to control *Mtb* infection directing the pathogenesis of disease. Diverse cytokines produced on *Mtb* infection determines the fate of host response. Cells of MPS recruited at the infection site trigger cascade of events necessary for the release of various pro-inflammatory cytokines such as IL-1β, IL-6, IL-12, TNF-α, and IL-18. Furthermore, the protective function of IL-1 during *Mtb* infection was first demonstrated in mice dually deficient in IL-1α/β or IL-1R1 signaling (87). Several findings have reported enhanced *Mtb* load and less survival of mice with defect in IL-1R1 signaling. By contrast, IL-1β and IL-18 are synthesized after processing by caspases-1 of pro-IL-1β and pro-IL-18, respectively. Besides caspase-1, four more caspases, caspases-11 and -12 of mouse and caspases-4 and -5 of human regulate the inflammatory processing. Inflammasome plays a decisive role in host defense, as mice lacking IL-1β, IL-1 receptor, or IL-18 was more prone to *Mtb* infection. Furthermore, ASC protein deficiency led to the severe form of disease in the murine model of TB. IL-1β production is known to rely on the early secreted antigenic target of 6 kDa (ESAT-6) secretion system 1 (ESX-1) of *Mtb*, which contributes to the expression of virulence genes encoded by region of difference (RD-1). Inflammasome formation mediated by ESX-1 relies on the host NLR family pyrin domain containing-3 (NLRP3) along with ASC protein (88). Based on the above observations, it can be speculated that failure of BCG to induce optimum protection in TB is attributed to the lack of IL-1β and IL-18 mediated by RD-1 (89).

We, therefore, postulate that adjunct therapy of BCG with innate ligands that can regulate inflammasome formation and can enhance its efficacy as a potential vaccine against *Mtb*. In addition, inflammasome regulates *Mtb* infection during early phase by activating innate immunity and also plays a decisive role in augmenting the adaptive immunity against the bacterium.

## Activation of CD4 T Cells and CD8 T Cells by MPS in Restricting *Mtb* Growth

The onset of the adaptive immune response to *Mtb* generally takes 8–11 days subsequent to primary exposure of *Mtb*. This involves the transportation of the bacterium to the draining lymph nodes (90). Infected MPCs are involved in the priming and proliferation of *Mtb*-specific effector T cells and their subsequent migration to the lungs.

T cells play a fundamental role in conferring defense against *Mtb*. IL-12 secreted by MPS is a crucial regulator of the differentiation of naive CD4 T cells to Th1 cells. IL-6, TGF-β, and IL-1β released by MPCs help in the differentiation to Th17 cells (91). Inflammasome generated by MPS in synergy with IL-6 facilitates Th17 cells development *via* upregulation of IRF4 and RORγt. In absence of TGF-β signaling, IL-1β coordinates with IL-6 and IL-23 to generate pathogenic Th17 cells (92). A concerted action of both Th1 cells and Th17 cells is essential to control *Mtb* infection. IFN-γ released by Th1 cells plays a fundamental role in the activation of MPCs and the release of TNF-α, a cytokine responsible for inhibiting the growth of *Mtb* (93). Recent evidences highlight the role of Th17 cells producing IL-17 and IL-22 in restricting *Mtb* (93). Th17 cells mainly recruit monocytes and Th1 cells to the lungs that help to clear infection rapidly (93). Recent studies showed that Tregs can effectively diminish Th1 immunity (94) or hinder the effector T cells influx to the lungs during initial phase of *Mtb* infection (95). Similarly, Th2 cells that secrete mainly IL-4 and IL-13 significantly contribute to the progression of TB (96).

Although, CD8 T cells sufficiently provide immunity against *Mtb*, but their role is not adequately studied as has been done in the case of CD4 T cells. However, the importance of CD8 T cells has been established by the fact that their depletion leads to higher susceptibility toward *Mtb* in the experimental model of TB. Furthermore, β_2_m-knockout mice died rapidly on exposure to *Mtb* (97). CD8 T cells released IFN-γ and granulysin lyse the *Mtb*-infected macrophages, as well as can induce perforin (Pfn)-mediated cytotoxicity to kill the *Mtb* (98, 99). Furthermore, CD8 T cells in lung can directly lyse *Mtb-*infected macrophages in a Pfn-dependent manner (100). In addition, the presence of CD8 T cells expressing granzyme B has been observed in the TB patients and latent infection (101). However, CD8 T cells secrete IL-17, TNF-α, IL-10, and IL-2 but IFN-γ is considered to be a key mediator in defense against *Mtb* (101). Furthermore, IFN-γ augments the production of various chemokines CXCL9, CXCL10, and CXCL11 and therefore helps in recruiting the cells toward granulomas. Recently, it has been shown that the *Mtb*-infected macrophages undergoing apoptosis releases several antigens in apoptotic vesicles, thus permitting the access of these apoptotic bodies to bystander cells to present antigen in context with MHC class I molecules. This can be confirmed by inhibiting the formation of blebbing in a plasma membrane by caspase inhibitors. Consequently, it may hamper the CD8 T cell activation. Furthermore, unconventional CD1-restricted γδ-TCR T cells can specifically respond to *Mtb* glycolipids (102). The γδ T cells are the less abundant type of T cells population, which differ from common αβ T cells in having gamma delta (γδ) glycoprotein chains bearing TCR on T cells (102). Mycobacterial phospho-antigens are known as the potent activators of Vγ9Vδ2 T cell functions (103). These cells recognize *Mtb*-infected monocytes and alveolar macrophages in a non-MHC restricted manner (104). γδ T cells are responsible for initiating defense mechanism upon *Mtb* infection by generating cytotoxic function, cytokine secretion, and contact-dependent lysis of infected cells (105).

## Involvement of Reactive Oxygen and Nitrogen Species in Controlling *Mtb* Infection

Antimicrobial ROS and RNI are critical in controlling *Mtb* infection. RNI and NO produced by MPCs are considered potent antimicrobial agents. Human alveolar macrophages can kill *Mtb* in an iNOs dependent manner. Whereas, macrophages obtained from healthy individuals that are latently infected with *Mtb* prevent the growth of bacterium by secreting NO (106). These results were validated in the murine model of TB, where the abrogation of inducible NO synthase activity resulted in a dramatic increase in the microbial burden (107). Moreover, disruption of *Inos* gene increases *Mtb* dissemination and mortality of the mice. Indeed, NO released by macrophages is critically dependent on IFN-γ (108). Although effector T cells are the key producer of IFN-γ but it takes few weeks for these cells to release IFN-γ. Nevertheless, the NO production by macrophages is noticed within 3 h of *Mtb* infection (109). NK and γδ T cells are the first to reach the infection site. They secrete IFN-γ that stimulates macrophages to produce NO. The antimycobacterial function of NO secreted by MPS is well documented in the case of mice. However, there are evidences that depict that NO secretion by human alveolar epithelial cells and macrophages inhibits the growth of *Mtb*, but the role of NO still needs to be fully authenticated in humans (110). Furthermore, NOS and NO are highly evident in the macrophages obtained from bronchoalveolar lavages of TB patients. Apparently, MDR patients secrete lesser NO, as compared with TB patients (111). In addition, it has been reported that NOS2-deficient mice are immunocompetent to cure *Mtb* infection (112). It has been demonstrated that ciprofloxacin elicits the release of NO to eliminate *Mtb* (113). Furthermore, NO regulates the secretion of many pro-inflammatory cytokines such as IL-1β, IL-8, and TNF-α (114).

However, *Mtb* has successfully developed immune evasion strategies to resist the intracellular killing by ROS and RNI produced by MPS. *Mtb* has phenolic glycolipid I, cyclopropanated mycolic acids, and LAM rich thick cell wall that is the effective scavenger of oxygen radicals providing resistance to ROS (115). Besides, *Mtb* produces various ROS scavenging enzymes such as KatG, superoxide dismutases (Sod A and C), peroxidase along with peroxynitrite reductase complex consisting of AhpC, AhpD, SucB (DlaT), and Lpd (116, 117). Interestingly, Lsr2 bound to *Mtb* DNA protects the pathogen from ROS mediated destruction (118). Truncated hemoglobin in *M. smegmatis* protects the bacterium from aerobic respiration by inhibiting the NO production (119). Surprisingly, despite the strong killing potential of RNI, ROS, and cytokines, *Mtb* has successfully learned to prevail in the host environment.

## MPS Resist *Mtb* Inflicted Death

The apoptosis (programmed cell death) is a well-known event, where a cell undergoing death still retains its cytoplasmic material within membranous vesicles called as apoptotic bodies. MPCs eliminate apoptotic bodies through the mechanism known as “efferocytosis,” which critically contributes in boosting host immune response. The caspase family of serine proteases is the essential molecules that generate apoptosis in MPS. Apoptosis operates through several classical pathways. One of the essential pathways is the ligation of TNF receptor family-2, which activates caspases and subsequently induces the formation of apoptotic bodies. Primarily, apoptosis occurs due to the nutrients deprivation, oxidative stress, or intracellular stresses that ultimately alter the mitochondrial membrane permeability. Consequently, cytochrome *c* is translocated to cytosol, which leads to the caspases activation. Another pathway is interceded by the release of granzyme B from cytotoxic T cells as well as NK cells. Subsequent to *Mtb* infection, MPCs augment the production of TNF-α, which elicits apoptosis. This process confines the growth of *Mtb* by activating local MPS and insulating it into the apoptotic vesicles. Intriguingly, considerable inhibition in *Mtb* growth was demonstrated when cells undergoing apoptosis were cocultured with naïve macrophages. In addition, it has been demonstrated that antimicrobial effect performed by macrophages is through the involvement of IL-1 signaling and NO-dependent anti-mycobacterial activity (120). The level of apoptosis induced by virulent and avirulent *Mtb* is quite distinct (121). Few reports signify that virulent strain of *Mtb* triggers necrosis by avoiding host defensive strategy, while avirulent strain induces apoptosis (121, 122). Furthermore, the frequency of apoptosis is quite high in the macrophages infected with an attenuated strain of *Mtb* (123). Even though the production of TNF-α is commensurate, MPCs infected with non-virulent *Mtb* are found to be more susceptible to undergo apoptosis. The possible reasons suggested for the differences between virulent and avirulent *Mtb* may be the virulence factors, bacterial load and duration of exposure. H37Rv infected MPCs secretes IL-10, leading to the induction of TNFR. The soluble form of TNF-α and TNFR complex inhibits the apoptosis (124). Furthermore, ESAT-6 of *Mtb*, leads to apoptosis in THP-1 macrophages by diminishing the expression of antiapoptotic molecules (125).

**Figure 1:**
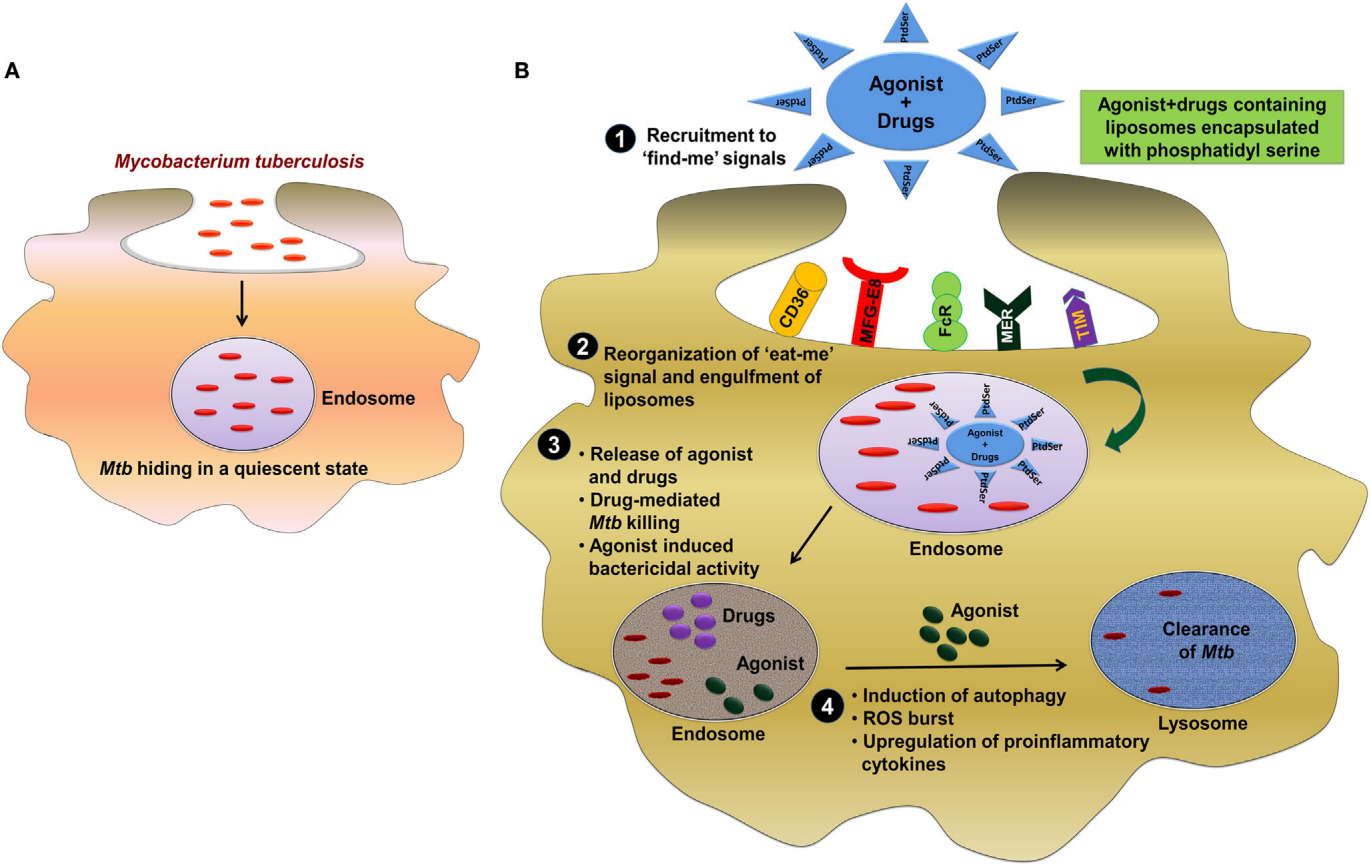
Involvement of “eat-me” signal in targeted delivery of immunomodulators along with anti-TB drug for the clearance of *Mtb*. **(A)** *Mtb* employs elegant strategy to impair the function of host cells by residing inside the endosome of macrophages in a quiescent state. **(B)** The most effective strategy to control and eliminate *Mtb* can be through targeting of pathogen-bearing MPCs by exploiting “eat-me” signal. One of the possible approach could be the engagement of phosphatidylserine (PtdSer) as an “eat-me” signal to deliver PRRs agonist/drug in liposome to target *Mtb* in the endosomes. (1, 2) Primarily, mononuclear phagocytic cells recognize “find-me” signal by several receptors such as CD36, MFG-E8, FcR, MER, TIM, and then phagocytose the liposomes through receptor-mediated endocytosis. (3) The direct clearance of *Mtb* in the endosome can be achieved by delivering the drug (rifampicin/isoniazid) to the site of infection. (4) However, the majority of *Mtb* would be eliminated but the eradication of residual bacterial population can be achieved by the agonist of TLRs (TLR-2, TLR-4, and TLR-9), NLRs (NOD-1 and NOD-2), and CLRs (Mincle, Dectin-1, and Dectin-2) mediated bactericidal mechanism and subsequently clearance of *Mtb* from lysosomes. Abbreviations: CD36, cluster of differentiation 36; MFG-E8, milk fat globule-EGF factor 8; FcR, Fc receptor; MER, membrane-bound receptor tyrosine kinase; TIM, T cell immunoglobulin and mucin domain; MPC, mononuclear phagocyte cell; NLR, NOD-like receptor; PtdSer, phosphatidylserine TLR, toll-like receptor; CLR, C-type lectin receptor; *Mtb, Mycobacterium tuberculosis*; TB, tuberculosis; NOD, nucleotide-binding oligomerization domain; Mincle, macrophage-inducible C-type lectin.

Neutrophil plays an imperative role in preventing *Mtb* infection (126). These cells reach first at the infection site. Furthermore, neutrophils phagocytose *Mtb* and generate ROS and restrict *Mtb* growth. In addition, they help macrophages to eliminate the infection. *Mtb*-infected neutrophils can also undergo apoptosis. At the time of apoptosis, these cells display “eat-me or find-me” signal on their plasma membrane, which helps in recognizing the unwanted constituents of MPCs (127). Macrophages recognize “find-me” signals and then phagocytose these cells. Macrophages engulf neutrophils undergoing apoptosis and secrete TNF-α to control *Mtb* infection by granulomas formation (128). It will be an exciting line of investigation employing the concept of “eat-me” signal to develop a novel strategy for targeted delivery of immunomodulators along with anti-TB drugs for the clearance of *Mtb* hiding in a quiescent state inside the endosome (Figure 1).

## Role of ER Stress (ERS) in the Regulation of Innate Immunity During *Mtb* Infection

In humans, endoplasmic reticulum (ER) performs various functions such as metabolism of lipids, protein folding, and maintaining cellular homeostasis. Different factors such as accumulation of unfolded proteins, loss of oxygen or hypoxia, and bacterial infections are responsible for the unfolded protein response (UPR), which causes ERS. Uncontrolled ERS leads to apoptosis. Furthermore, UPR activates various innate signaling pathways, which result in the survival of intracellular pathogens such as *Mtb* (129, 130). Apoptosis of macrophages helps to prevent the spread of mycobacterial infection by activating innate immunity (131). However, growing number of findings suggest that *Mtb* has evolved various strategies to control the ERS for its survival in the host (132). *Mtb* can efficiently alter the structure of macrophage ER. It was shown that macrophages infected by virulent (H37Rv) along with avirulent (H37Ra) *Mtb* strains possess distinct ER phenotypes (133). Difference in the morphology of ER in macrophages targeted during *Mtb* infection is a crucial factor for initiation of apoptosis. Ca^2+^ is very important in different apoptotic pathways and is responsible for the phagosome–lysosome fusion. A smooth ER phenotype linked with avirulent *Mtb*-infected macrophages increases cytosolic Ca^2+^ levels and simultaneously increases the synthesis of phosphatidyl choline/phosphatidyl ethanolamine (PC/PE), which leads to apoptosis. However, H37Rv but not H37Ra manipulates rough ER of macrophages and disturbs the cholesterol homeostasis to inhibit the apoptosis and establishes its intracellular persistence.

Endoplasmic reticulum stress is already known to influence macrophages. It stimulates conversion of macrophages toward M1 phenotype and induces apoptosis, thereby aiding in *Mtb* clearance. On the other hand, polarization of M2 phenotype by *Mtb* infection aids its escape by suppressing apoptosis (134). At the site of granuloma formation during *Mtb* infection, ERS markers such as activating transcription factor-3, pIre1α, and eukaryotic initiation factor 2α levels are increased (135). Depending on ERS, macrophage apoptosis is influenced by various *Mtb* proteins such as ESAT-6, 38-kDa antigen and PE-PGRS33. Henceforth, ERS is crucial in imparting protection against *Mtb* and restricting it to advanced granulomas (63, 136).

## MPCs Restrict the *Mtb*-Induced Inhibition of Phagosome Maturation

Mononuclear phagocyte cells have developed an array of strategies to control *Mtb* infection. MPCs recognize and phagocytose *Mtb* through PRRs such as C-type lectins, Dectin-1, Dectin-2, Mincle, macrophage C-type lectin, DC-SIGN, MR, scavenger receptors-A, and macrophage receptor with collagenous structure (MARCO) (scavenger receptors) (137). Consequently, activation through these receptors triggers various downstream signaling pathways mainly through Rac1–2, Cdc42, and most importantly GTPases. Arp2/3 is a key activator of actin polymerization. It is a primary step in instigating the process of phagocytosis and is triggered by the interaction of Wiskott–Aldrich syndrome protein with Rac1–2 and Cdc42 (43). Phagocytosis of *Mtb* triggers the formation and maturation of phagosome. During this process, a sequence of events occurs involving several molecules (25). Fc-gamma receptor along with MR is involved in the antigen trafficking to early phagosome. Early phagosome formation occurs upon interaction of MR with *Mtb* lipids such as PIMs and manLAM (138). The phosphorylation of immunoreceptor tyrosine-based activation motif by kinase of Src family, followed by downstream phosphorylation of Src homology region 2 domain-containing phosphatase-1 (SHP-1) and ras-related C3 botulinum toxin substrate (RAC) are critical for phagosome maturation (139). Membrane molecules exchange and deliver cargo either by “touch and run” or complete fusion with phagosome undergoing maturation. Motor proteins such as dynein and dynactin are key players to bring vesicles in an appropriate orientation for vesicular fusion, which is important for phagosome maturation. Many SNARE proteins such as vesicle-associated membrane proteins-7 and VAMP-8 are also involved in this event. During phagosome maturation, early endosome carrying *Mtb* undergo closure forming phagocytic cup by various coat proteins such as coronin or tryptophane aspartate-containing coat protein (140). Recruitment of proteins such as PX or FYVE motif proteins such as early endosome antigen 1 (EEA1) to early endosome for phagosome maturation is done through phosphotidylinositol-3-phosphate (PI(3)P) (141, 142). Endosome fusion to phagosome leads to oxidative and hydrolytic environment, which ultimately causes cargo degradation (143). Recruitment of lysosome-associated membrane proteins (LAMPs) 1 and 2 is a characteristic feature of late endosomal stage. Acidic pH of around 5 is an important marker of the late endosomal stage to control *Mtb* growth. This acidification process is controlled by Abl tyrosine kinase that functions as a negative regulator of phagosome maturation. Inhibition of this kinase by certain drugs such as imatinib results in controlling *Mtb* growth (144). The lipid body formation in the cell is induced by various bacterial infections such as *S. aureus, M. leprae*, and *Mtb*. The fusion of lipid compartments of the cells has been shown to be important for the maturation of phagosomes containing *Mtb* (145). However, *Mtb* resist this process of eradication by interfering in the maturation of phagosomes and subsequent fusion with lysosome (44). *Mtb* bearing phagosomes show reduced acidification due to halt H^+^-ATPase (146). The lipids produced by *Mtb* inside the macrophages mimic the host lipids such as phosphatidylinositol (PI3P) giving rise to inhibition of PI3P trafficking (147). EEA1 is an important molecule that inhibits Rab5 and acts as a key player in membrane fusion (148). There is a reduced recruitment of EEA1 in *Mtb-*infected macrophages, which inhibits the maturation of phagosome (149). Ca^2+^ is an important molecule involved in the phagosome maturation. However, *Mtb* inhibits sphingosine kinase dependent Ca^2+^ increase, leading to reduced phagosome maturation (150).

**Figure 2:**
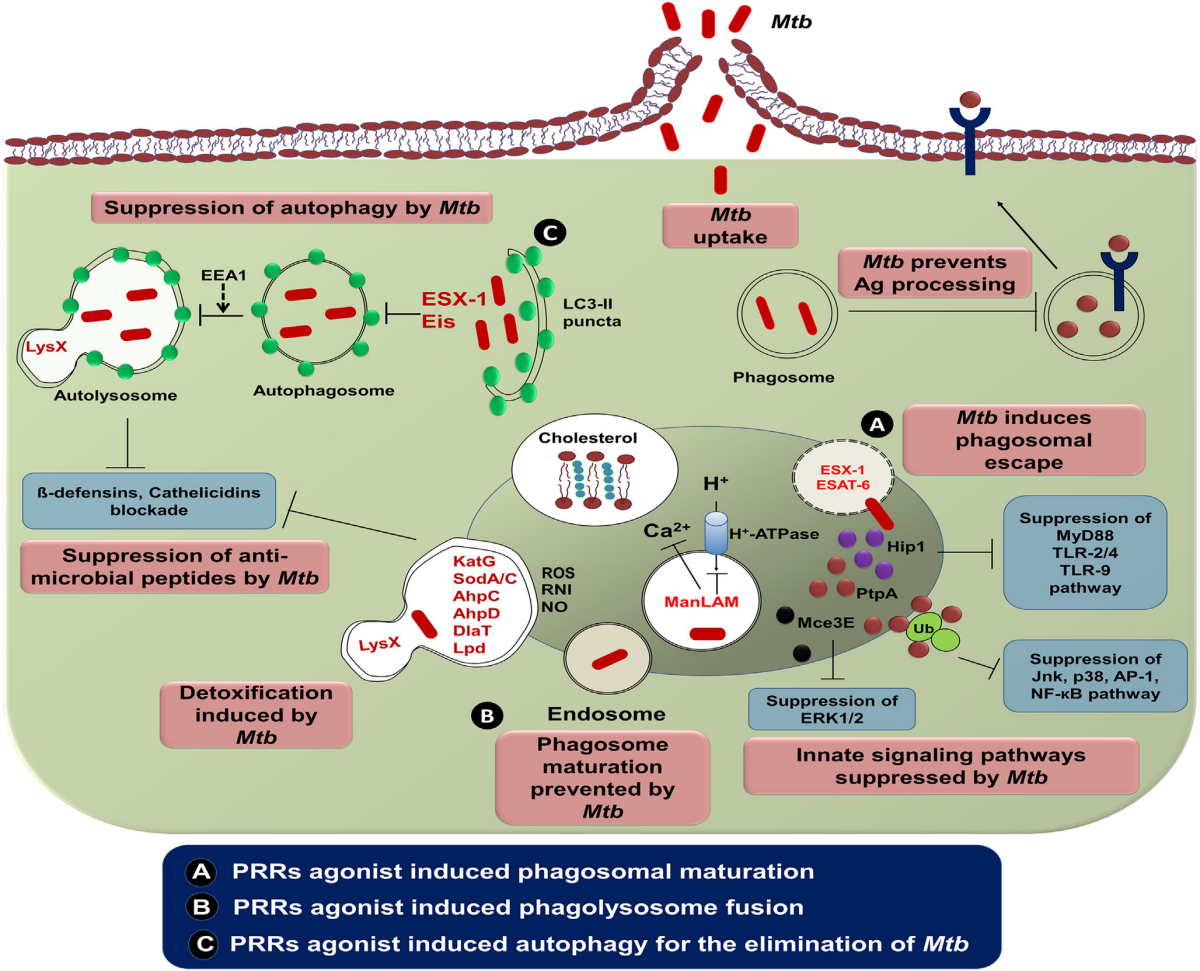
The intracellular evasion strategies adopted by *Mycobacterium tuberculosis* (*Mtb*) and its counteraction through cellular defense mechanism. Phagocytosis of *Mtb* is promoted by diverse cell-surface receptors and cholesterol present in the mononuclear phagocytic cells. *Mtb* utilizes the host cholesterol for its survival and impedes antigen processing and presentation by its lipoproteins. Consequently, ESAT-6 and ESX-1 of *Mtb* alter phagosome maturation process. The potential virulence factors, namely, PtpA and Mce3E of *Mtb* ultimately restrain various signaling cascades of innate immunity by binding with host ubiquitin. Another virulent factor of *Mtb*, ManLAM arrests phagosomal maturation *via* interrupting the transport of host H^+^-ATPase to phagosomes and blockading cytosolic Ca^2+^ release. *Mtb* enzymes such as KatG, SodA/C, NADH-dependent peroxidase, superoxide dismutases, and DlaT are involved in detoxification of ROI and RNI. Neutralization of antimicrobial peptides is accomplished through mycobacterial protein LysX. Suppression of autophagy in mononuclear cells is rendered by the *Mtb* encoded gene “enhanced intracellular survival (Eis).” **(A)** Several PRRs agonist such as TLRs (TLR-2, -4, and -9), NLRs (NOD-1 and NOD-2), and CLRs (Mincle, Dectin-1, and Dectin-2) induce phagosomal maturation and inhibit *Mtb* growth by membrane cholesterol reduction. **(B,C)** Involvement of these agonists triggers the phagolysosome fusion and subsequent process of autophagy. To monitor the effect of targeting various PRRs, a comprehensive investigation is required, before selecting the best combination of agonists to control *Mtb* infection. Abbreviations: Hip1, huntingtin-interacting protein 1; PtpA, protein tyrosine phosphatase A; Mce3E, mammalian cell entry operon 3E; ManLAM, mannose lipoarabinomannan; EEA1, early endosome antigen 1; ESAT-6, early secreted antigenic target of 6 kDa; ESX-1, ESTAT6 secretion system l; LysX, lysylphosphatidylglycerol biosynthesis bifunctional protein; KatG, catalase-peroxidase; SodA/C, superoxide dismutase A/C; AhpC/D, alkyl hydroperoxide reductase subunit C/D; DlaT, dihydrolipoamide acyltransferase; Lpd, lipoamide dehydrogenase; Ag, antigen; Ub, ubiquitin; MyD88, myeloid differentiation primary response gene 88; TLR, toll-like receptor; Jnk, c-Jun N-terminal kinase; AP-1, activator protein 1; NF-κB, nuclear factor-κB; ERK1/2, extracellular signal-regulated protein kinases 1 and 2; LC3, microtubule-associated protein 1A/1B-light chain 3; ROS, reactive oxygen species; RNI, reactive nitrogen intermediates; NO, nitric oxide; CLR, C-type lectin receptor; NOD, nucleotide-binding oligomerization domain; Mincle, macrophage-inducible C-type lectin.

Phagosome containing *Mtb* express abnormal early endosomal markers for instance small GTPase Rab5, transferrin along with its receptor and absence of late endosomal markers *viz* small GTPase Rab7 along with vacuolar proton transporter v-ATPase (146). Furthermore, there is a reduced level of PI3P, EEA1 along with hepatocyte growth factor–regulated tyrosine kinase substrate (HRS) onto the *Mtb* phagosome membrane. These molecules are implicated in the protein sorting and fusion of the phagosome with late endosome followed by lysosome (151). Recently, it has been demonstrated that protein tyrosine phosphatase A (PtpA) binds to one of the subunits of v-ATPase leading to dephosphorylation of vacuolar-sorting protein 33B (152). Furthermore, *Mtb* glycolipids such as LAM as well as TDM inhibit the phagolysosome fusion (153). *Mtb* employs its type VII secretion system by exporting effector proteins EsxH and EsxG to destroy endosomal sorting complex required for transport and thereby impairing the maturation of phagosome (154). Overall, this signifies that the modulation of phagosome maturation of MPS can ultimately lead to the eradication of *Mtb*. In future, it can be used as an important therapeutic platform to control TB (Figure 2).

## Regulatory Role of MPCs in Inducing Autophagy Against *Mtb*

Autophagy is an intracellular degradation phenomenon that is developed during the stress response. It allows cells to alter their biomass and turn over components during starvation. Autophagy specifically targets the cytoplasmic components, which include organelles, macromolecules, and cells undergoing unintended cell death to lysosomes for their degradation. It ultimately leads to a periodical cleaning of the cell interiors. Similarly, autophagy has an essential role in numerous diseases, which includes cancer, degenerative diseases, as well as aging. In addition, autophagy augments the ability of cells to engulf and eliminate microbes and thereby protects the host. Treatment with rapamycin or IFN-γ or starvation can initiate and enhance autophagy. Furthermore, signaling through PRRs has been reported to have direct association with the induction of autophagy. It has been well established that triggering through various agonists of TLR-3, TLR-4, and TLR-7 can promote autophagy (155).

Autophagy boosts bactericidal mechanism by sequestering of the process in “double membrane envelope” structure called autophagosome. These processes follow the fusion of autophagosome with lysosomes by forming autolysosome for the subsequent elimination of the mycobacterium. Autophagy can be initiated within 30 min, as shown through the conversion of LC3-I to LC3-II, which is a fundamental indicator of this process. Autophagy helps in the clearance of *Mycobacterium bovis* BCG as well as *Mtb* by transporting them to the lysosome for their successive degradation (156). It has been reported that triggering infected macrophages through TLR-7 stimulates autophagy and can curb the intracellular growth of *Mtb* (155). Autophagy not only transports *Mtb* to lysosomes but also delivers bactericidal components to the *Mtb* degradation compartment. This observation was further confirmed by blocking the autophagy through knockdown of *Atg5* and *Beclin 1*. Both these molecules are considered to be essential for autophagy. It may be an exciting line of study to identify the mechanism that triggers the autophagy-mediated clearance of *Mtb*. One of the possible mechanisms is an ubiquitination process, where the arrangement of “poly-ubiquitinylated protein aggregates” and contributes as an autophagy substrate. These protein aggregates are broken down into bactericidal peptides, which contribute in destroying *Mtb* (157).

*Mycobacterium tuberculosis* impedes MPCs bactericidal mechanism by deactivating the acidification of phagosome, lysosome and subsequently inhibiting the phagosome–lysosome fusion. Interestingly, autophagy directs the innate immunity to obstruct the evasion strategies adopted by *Mtb* by targeting the bacterium inhabiting inside, as well as outside the phagosome (156, 158). Similarly, BCG also hampers fusion of the phagosome with the lysosome, consequently resulting in the interference of antigen processing, presentation and therefore impairment in T cell response. This is considered as one of the possible reasons for BCG failure to safeguard people living in TB-endemic regions.

**Figure 3:**
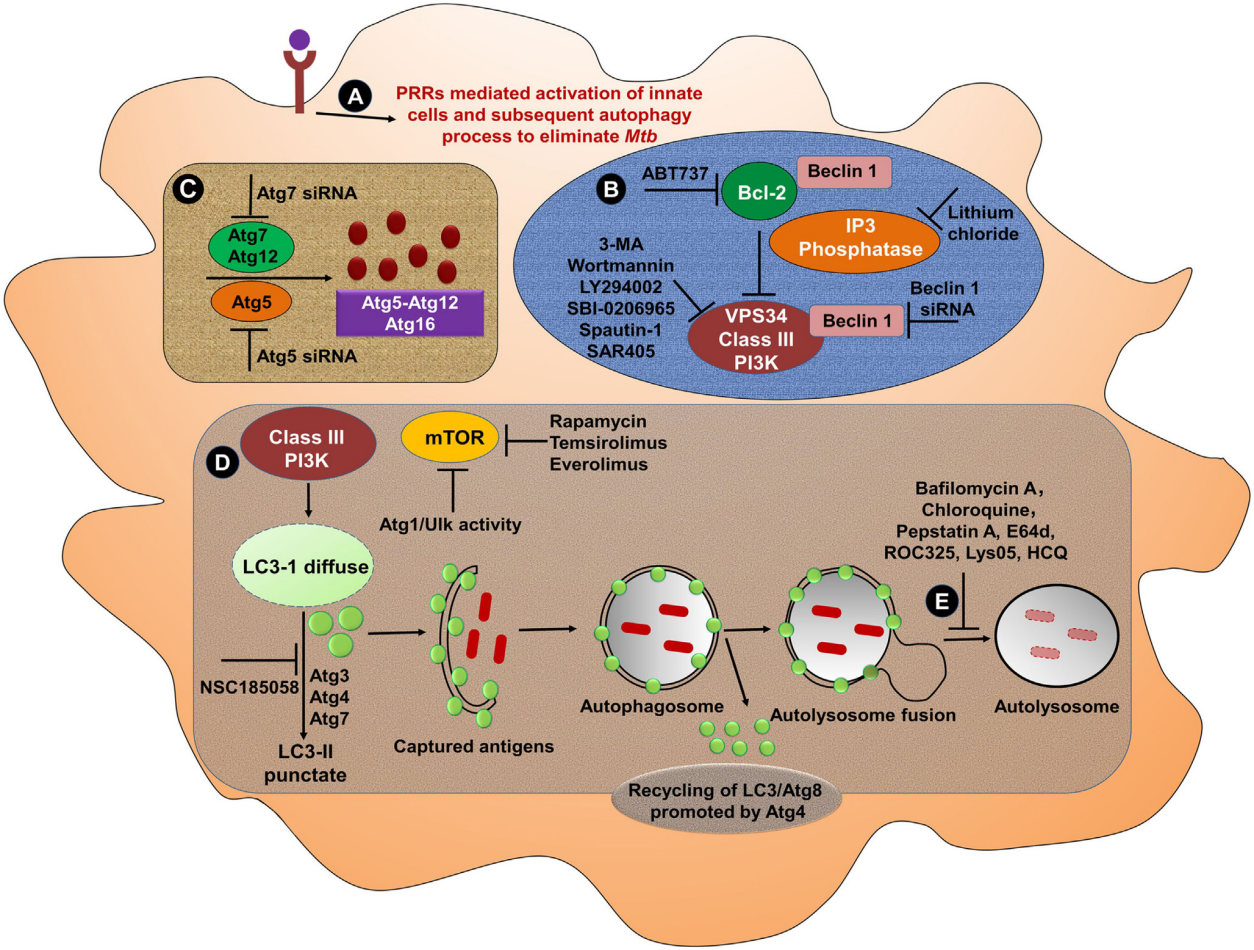
Bolstering the functionality of mononuclear phagocytic cells through PRRs and induction of autophagy. **(A)** Several PRRs-mediated approaches can be used to activate MPS. Activated cells then undergo autophagy to eliminate *Mtb*. Various strategies can be employed to induce or block different steps of autophagy from phagosome maturation to lysosomal fusion. **(B)** At the initiation stage of autophagy, the phagophore formation or nucleation processes occur. One of the most frequently used genetic approaches to inhibit autophagy is knockdown or knockout of *Beclin 1* gene to demonstrate the specificity. The activation of *Beclin 1* gene induces autophagy. The instigation of autophagy can be originated through the activation of PI3 kinase pathway. Treatment of cells with 3-MA, wortmannin, LY294002, SBI-0206965, spautin-1, and SAR405 inhibits the activity of class III PI3K for instance Vps34, which abrogates autophagy process. Autophagy can be artificially induced using lithium chloride, which inhibits inositol phosphatase, leading to augmented intracellular PI3P levels. Other targeted peptides, such as ABT737 that obstruct the interaction of Beclin 1 with Bcl-2 have also been validated to promote autophagy (160). **(C)** Similarly, the knockdown of *Beclin 1* can effectively inhibit the autophagy, leading to the knockdown of *Atg5* (161). Therefore, the knockdown of *Beclin 1* may be the preferred approach to inhibit the autophagy. **(D)** The treatment with rapamycin induces the autophagy through its capacity to obstruct the inhibitory activity of mTOR. Subsequently, the conversion of LC3-I to LC3-II, capturing antigens and phagosome–lysosome fusion can effectively clear pathogens. **(E)** Several inhibitors such as bafilomycin A1 that inhibit the lysosomal Na^+^H^+^ ATPase are frequently used to reduce lysosomal turnover of autophagosomes. Other agents such as chloroquine, HCQ, Lys05, and ROC325 increase pH, lead to the prevention of the lysosomal acid proteases, as well as cause autophagosomes to accumulate in the lysosome (162, 163). The specific inhibitors of lysosomal proteases, for instance, pepstatin A or E64d abrogates the autophagy (164). Abbreviations: PRRs, pattern recognition receptors; Atg, autophagy-related protein; siRNA, small interfering RNA; Ulk, Unc-51-like kinase 1; ROC325, inhibitor of lysosomal-mediated autophagy; Lys05, dimeric chloroquine (lysosomal autophagy inhibitor); 3-MA, 3-methyl adenine; LC3, microtubule-associated protein 1A/1B-light chain 3; PIK3C3/Vps34, class III phosphatidylinositol-3-kinase; SAR405, selective ATP-competitive inhibitor of Vps34; HCQ, hydroxychloroquine; E64d, ethyl-ester of E64c; Bcl-2, B cell lymphoma-2; ABT-737, BH3 mimetic inhibitor of Bcl-2; NSC185058, inhibitor of Atg4B; mTOR, mammalian target of rapamycin; ATP, adenosine-5′-triphosphate; MPS, mononuclear phagocyte system; *Mtb, Mycobacterium tuberculosis*.

By contrast, autophagy in MPCs promotes both *Mtb* and BCG antigens processing and presentation. Mice adoptively transferred BCG infected APCs that were incubated with rapamycin, elicited Th1 cells that protected against *Mtb*. Rapamycin-induced autophagy with subsequent antigen processing and presentation was suppressed by treatment of cells with the autophagy inhibitors 3-MA or small interfering RNA against *Beclin 1* (159). Designing of a novel approach with an appropriate adjuvant to induce autophagy may be an alternative and effective strategy to make BCG an effective vaccine for people living in TB-endemic zones. To achieve a full pharmacological evidence of the importance of the phagocytotic process in TB, it will be of interest to ascertain whether several PRR agonists or certain autophagy inducers are capable of stimulating the formation of autophagolysosome in MPCs (Figure 3).

## Development of the Possible Immunotherapeutic Strategies to Enhance Anti-TB Immunity

After the discovery of anti-TB drugs, it was assumed that the disease can be easily eliminated. Unfortunately, this could not be achieved due to the lengthy regimen, narrow therapeutic index and emergence of drug-resistant strains of *Mtb* (165). Currently, novel therapies are being explored for the treatment of numerous ailments such as cardiac diseases, cancer, and autoimmunity. In recent times, better information of host–pathogen interplay has given rise to a paradigm shift in remedial measures such as host-directed therapies, signaling pathway blockade, stem cells, signaling *via* receptors, adoptive transfer of antigen-loaded DCs to protect against cancers, treatment with immunomodulators and humanized Abs, probiotics as well as herbal remedies. In addition, Food and Drug Administration has permitted anti-cytotoxic T lymphocyte-associated antigen-4, CD80 as well as CD52 antibodies for treating cancer. Besides this, interferon-β is endorsed for the treatment of multiple sclerosis (166, 167). In spite of promising immunotherapies in diverse diseases, no thoughtful effort has been attempted in case of TB. Furthermore, immunotherapies with agonists of PRRs in conjunction with drugs have shown to improve the clinical outcome of the disease (168, 169). It will be of great interest to monitor the impact of drugs on *Mtb* in association with the immunomodulatory activity driven through PRRs. Such stratagem has dual advantage over the treatment with drugs alone. The drug will kill the bacterium residing in the MPCs, whereas immunomodulators will stimulate the host immunity to eliminate the pathogen, which had escaped the killing by the drug. Furthermore, this approach may not only reduce the dose as well as duration of the anti-TB drug regimen but can also curb the development of drug resistance in *Mtb*. Therefore, in future, immunotherapies may be the best choice to treat TB and its drug-resistant form.

The second most effective strategy to control and eliminate any disease is vaccine (3, 4). Presently, BCG is the only existing vaccine to treat TB. Ironically, BCG is the most controversial vaccine because of its highly variable efficacy worldwide. Moreover, it protects only children but not adults, as very categorically evident by 15 years follow-up study in India (170). The urgent necessity and challenge for the scientific communal is to improve the current drug regimen or develop alternative and innovative stratagems against TB. In essence, reinforcing the immunity against *Mtb* by triggering through the receptors of innate immunity might be a prudent idea to treat TB patients.

## Conclusion and Future Prospects

There is no iota of doubt that for several years TB is treated with potent drugs. Unfortunately, the disease is neither controlled nor eradicated by these drugs; rather the regime has contributed in gifting resistant strains of the *Mtb*. Consequently, it is an important to devise and discover innovative and alternative therapies to control TB. In this connection, understanding the struggle between the bacterium and the host cells such as MPS may have an important impact on the disease outcome. The safe survival heaven for *Mtb* is MPCs. Targeting molecules such as PRRs that can optimally elicit MPCs to eliminate *Mtb* will be an interesting strategy to employ as an adjunct therapy along with the anti-TB drugs. There may be a distinct possibility of such therapeutic measurement in controlling TB by decreasing the dose and duration of drugs and also curbing the emergence of mono and MDR strains of the bacterium. In this article, we have mentioned about a possible combination of various PRRs such as TLRs (TLR-2, TLR-4, and TLR-9), NLRs (NOD-1 and NOD-2), and CLRs (Mincle, Dectin-1, and Dectin-2) as suggested by several studies, to control *Mtb* infection. However, studies are still required to select the best possible combination of PRRs to achieve complete elimination of *Mtb*. Exploration of this strategy may be an exciting line of future study to control TB.

## Author Contributions

In this manuscript, the concept and theme were generated by JA and SP. The writing of manuscript and figures were done by SP, GK, SN, MA, DD, HB, SS, MN, JK, and JA.

## Conflict of Interest Statement

The authors declare that the research was conducted in the absence of any commercial or financial relationships that could be construed as a potential conflict of interest.
